# Cyclosporine and Vancomycin + Amikacin Induced Hot Kidney Appearance in a Young Adult and a Pediatric Patient

**DOI:** 10.4274/mirt.30592

**Published:** 2017-10-02

**Authors:** Derya Çayır, Mine Araz, Mustafa Filik, Mehmet Erdoğan

**Affiliations:** 1 University of Health Sciences, Dışkapı Yıldırım Beyazıt Training and Research Hospital, Clinic of Nuclear Medicine, Ankara, Turkey

**Keywords:** kidney, Tc-99m medronate, Radionuclide imaging

## Abstract

The appearance of a hot kidney on bone scintigraphy is rare and can be seen due to various factors. In our clinic, we observed hot kidney appearance in two patients to whom technetium-99m methylene diphosphonate (Tc-99m MDP) whole body scan has been performed: a young male adult at the age of 18 who was diagnosed with acute lymphocytic leukemia with a presumptive diagnosis of avascular necrosis, and a 9-year-old girl with cystitis for a pre-diagnosis of osteomyelitis. The first patient had a history of cyclosporine usage and the second patient was being treated with amikacin + vancomycin. To the best of our knowledge, we present the first cases where hot-kidney appearance on Tc-99m MDP whole body scan due to the use of cyclosporin and amikacin + vancomycin is demonstrated.

## Abstract (tr)

Kemik sintigrafisinde hot kidney görünümü nadir olup, çeşitli faktörlere bağlı olarak görülebilmektedir. Kliniğimizde teknesyum-99m metilen difosfanat (Tc-99m MDP) tüm vücut kemik sintigrafisi yaptığımız iki hastada; ilki avasküler nekroz ön tanısı ile 18 yaşında akut lenfositer lösemi tanılı genç erişkin erkekte, diğeri osteomiyelit ön tanısı ile 9 yaşında sistit tanılı kız çocuğunda hot kidney görünümü izledik. İlk hastada siklosporin kullanım öyküsü vardı, ikinci hasta ise amikasin + vankomisin tedavisi altındaydı. Bildiğimiz kadarıyla, Tc-99m MDP tüm vücut kemik sintigrafisinde siklosporin ve amikasin + vankomisin kullanımına bağlı hot kidney görünümünün gösterildiği ilk olguları sunuyoruz.

## Figures and Tables

**Figure 1:**
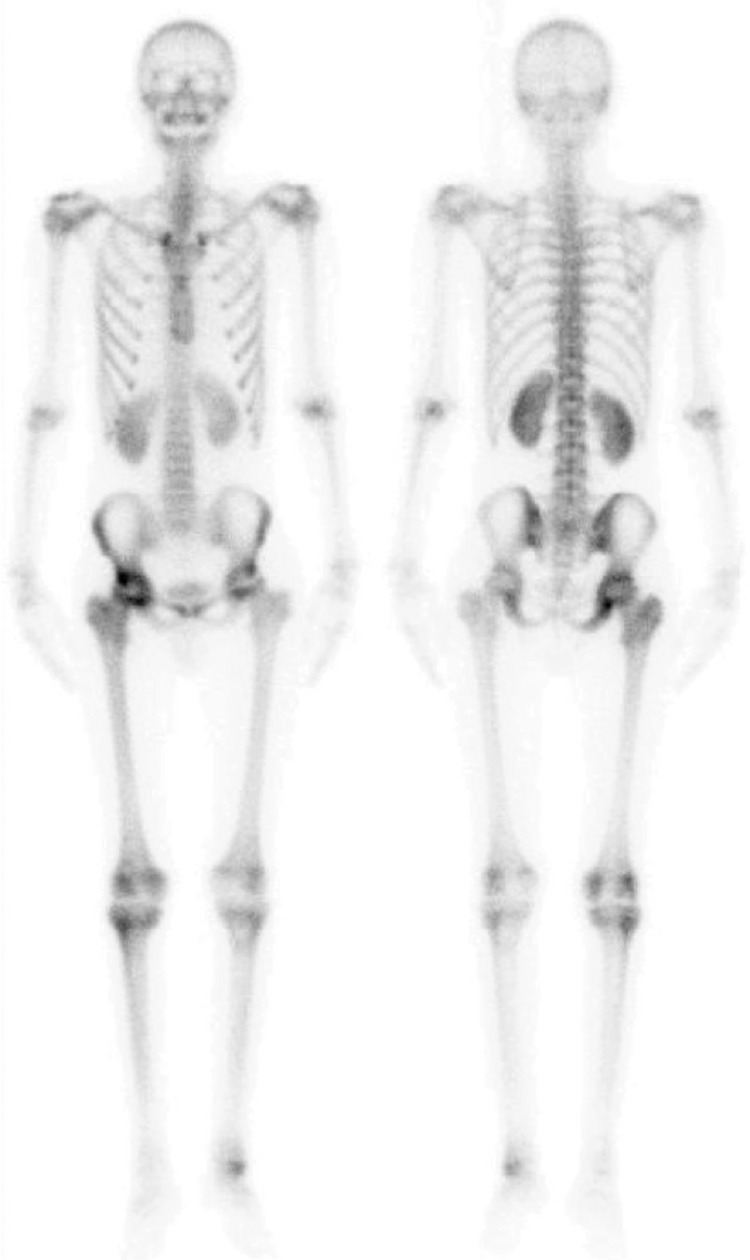
A male patient at the age of 18 who has been diagnosed with acute lymphocytic leukemia was under cyclosporine treatment for 9 months. The patient suffered from hip and right leg pain, and Tc-99m MDP whole-body bone scintigraphy was performed for possible avascular necrosis. There was no pathological finding throughout the skeleton except for increased peripheral osteoblastic activity in the middle of the right femur, hypoactive area in the middle, and mildly increased activity in the femur neck and trochanteric region, findings in accordance with the preliminary diagnosis. As an additional finding, diffuse increased activity was observed in both kidneys. Abdominal ultrasonography performed before immunosuppressive therapy revealed that both kidneys were normal. Following cyclosporine treatment, serum urea level raised to 42 mg/dL (normal range: 11-39 mg/dL) and serum creatinine level was detected as 1.23 mg/dL (normal range: 0.5-1.2 mg/dL). Urinary ultrasonography showed bilateral grade 1 increase in renal parenchymal echogenicity

**Figure 2:**
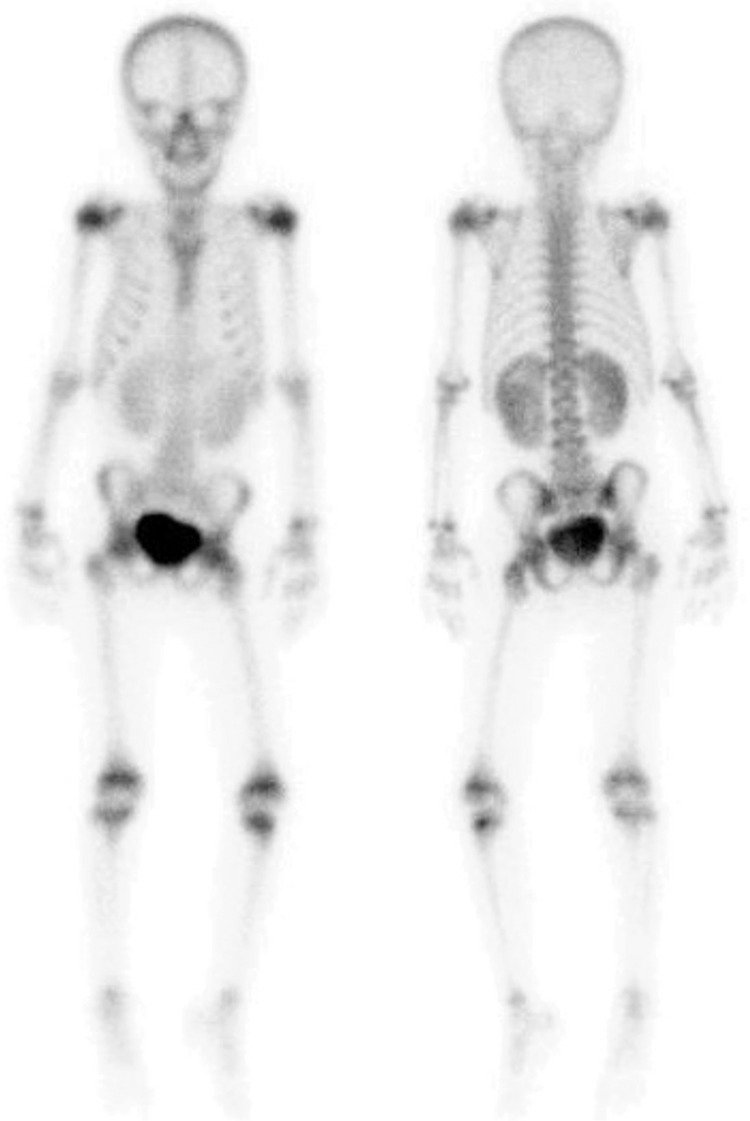
A 9-year-old girl with a diagnosis of cystitis has been receiving amikacin and vancomycin treatment for a week. She had persistent fever, fatigue and widespread body pain despite normal urinary US findings, and normal blood urea nitrogen and creatinine levels. Tc-99m MDP whole body scintigraphy was performed to rule out osteomyelitis. Scintigraphic findings were normal, but both kidneys were enlarged and showed diffuse increased radiopharmaceutical uptake. Tc-99m MDP is a highly sensitive method for assessing dissemination of primary skeletal system disorders (1). Tc-99m MDP uptake in the soft tissue can be detected due to various reasons. Diffuse increased radionuclide uptake in the kidneys is defined as ‘hot kidneys’. The incidence of hot kidneys on bone scintigraphy was reported to be less than 1% (2). There are several proposed reasons for bilateral diffuse increased kidney uptake on Tc-99m MDP bone scan. It has been suggested that renal damage causes deterioration of secretory or glomerular filtration function (3). Another mechanism of Tc-99m MDP uptake can be calcification of the kidneys due to ischemia caused by injury to the kidney at any time (3,4). The common causes of the appearance of hot kidneys include nephrotoxic drugs (antibiotics, chemotherapeutics, and nonsteroidal anti-inflammatory agents), urinary obstruction, nephrocalcinosis, metastatic calcification, hypercalcemia, hyperparathyroidism, infective pyelonephritis, vascular pathologies, iron overload, radiotherapy, and rhabdomyolysis (3,4,5,6,7,8,9,10,11,12,13,14,15,16). We observed the appearance of “hot kidneys” in our two cases, which may be due to temporary renal damage secondary to the long-term use of cyclosporine and amikacin + vancomycin. Cyclosporin is a calcineurin inhibitor that provides immunosuppression by blocking the production of interleukin-2 by T cells. Cyclosporine reduces glomerular filtration rate by causing vasoconstriction in afferent arterioles in the kidneys (17,18). Aminoglycosides (amikacin) are used in the short-term treatment of infections caused by susceptible strains of gram-negative microorganisms. Vancomycin is used in the treatment of infections caused by gram-positive microorganisms. Nephrotoxicity can be seen in the use of these two drugs in combination, especially in long-term or high-dose use. The glomerular filtration rate may be reduced and the nephrotoxic effect may be caused also by impaired proximal tubular transport. Tc-99m MDP might sometimes show extra-skeletal uptake in the soft tissue. The urinary system is mildly visualized on a normal bone scan, but symmetrical diffuse increased Tc-99m MDP uptake is almost always pathological and should be mentioned in the reports and etiology should be further investigated
